# CornPheno: Phenotyping corn ear kernels in the wild via point query transformer

**DOI:** 10.1016/j.plaphe.2025.100129

**Published:** 2025-10-15

**Authors:** Xin Li, Pinzhe Li, Xinzhe Wang, Yaning Zhu, Tianqi Hu, Binghui Xu, Yongshuai Zhang, Zhiguo Han, Hao Lu

**Affiliations:** a National Key Laboratory of Multispectral Information Intelligent Processing Technology, School of Artificial Intelligence and Automation, Huazhong University of Science and Technology, Wuhan, 430074, China; b School of Mechanical Science and Engineering, Huazhong University of Science and Technology, Wuhan, 430074, China; c PhenoTrait Technology Co., Ltd., Beijing, 100096, China; d MetaPheno Laboratory, Shanghai, 201114, China

**Keywords:** Plant phenotyping, Corn ears, Corn kernels, Field-based phenotyping, Plant counting

## Abstract

Corn is a globally important economic crop. Certain trait parameters of corn ears kernels per ear are essential indicators for corn breeding. However, acquiring these parameters faces two challenges: i) manual measurement is labor-intensive and error-prone, and ii) vision-based corn phenotyping machines require fixed image capturing environment and are cost-prohibitive. To address these limitations, we introduce CornPheno, a user-friendly, low-end, smartphone-based approach capable of executing corn ear phenotyping in the wild. CornPheno highlights three corn ear parameters: kernels per ear, rows per ear, and kernels per row. Technically, inspired by crowd localization in computer vision, we first extract kernels per ear based on a Corn data-trained Point quEry Transformer (CornPET). CornPET generates interpretable per-kernel point predictions and supports subsequent row detection. To detect rows, we introduce a novel point-based corn row detection approach, termed unicorn, featured by sqUeezed clusteriNg and bI-direCtional pOint seaRchiNg, to phenotype rows per ear and kernels per row. With adaptive geometric modeling, our approach is robust to partial rows, curved rows, and missing kernels. To promote the use of CornPheno, we have integrated it into OpenPheno, a WeChat-based mini-program, and made it open-access for corn breeders. We hope our approach can provide the community with a user-friendly and cost-effective way to facilitate corn breeding.

## 1 Introduction

Corn (*Zea mays*) is one of the three major food crops, cultivated worldwide, and is the top food crop globally [1,2]. In China, corn production accounted for 41 ​% of the total crop production (28.9 million tons) in 2023. The corn planting area comprised 37 ​% of the total crop planting area, reaching 44.2 million hectares [1,[3], [4], [5]]. Due to its high nutrient and protein content [6], corn serves as a raw material for commodities like starch, flour, and ethanol, and as the main feedstock for livestock, including pigs and cattle [[7], [8], [9]]. For global food security, corn has undergone significant breeding evolution.

Throughout corn breeding, a number of traits are required for selecting superior varieties such as kernels per ear, rows per ear, and kernels per row. They are typically obtained by phenotyping corn ears and are related to the grain yield, stress resistance, and cultivation, finally used by plant breeders to choose a certain cultivar [[10], [11], [12], [13]]. During each growing season, breeders cultivate approximately 40, 000 corn plants per hectare, phenotype the traits of each plant, and retain the top two planting rows (∼5 ​%) [14]. This breeding loop involves a great deal of repetitive labor [15,16].

Recently, artificial intelligence (AI) technologies have been increasingly applied to plant phenotyping to reduce manual labor and errors. Specifically, certain corn breeding machines employ image processing and computer vision to extract corn trait parameters. However, they incur expensive costs due to customized imaging systems and mechanical parts. Additionally, the need for a fixed image-capturing environment restricts their outdoor use. To expedite corn breeding, two challenges must be addressed: i) creating a robust approach applicable to complex environments; ii) capturing images efficiently for high-throughput demand.

Previous studies have explored alternatives to corn ear breeding machines. Makanza et al. [17] proposed a field-based corn image phenotyping method, but necessitates professional camera equipment and manual placement of corns on plain black backgrounds. Wu et al. [18] proposed automatic kernel counting using RGB images, requiring a blue background for accurate image segmentation. Gillette et al. [19] proposed an approach utilizing smartphone-captured images, but the smartphone needs to be placed at a fixed distance with stable lighting conditions. Although machine-based image capturing is not required, these methods do not showcase sufficient robustness in complex environments, especially when lighting and background vary.

To tackle these challenges, we present CornPheno, a user-friendly, low-cost smartphone solution for in-situ corn ear phenotyping in open environments. We focus on three quantity-related parameters: kernels per ear, rows per ear, and kernels per row. By recognizing the similarity between counting kernels and counting crowd, we repurpose our previously developed crowd localization model Point quEry Transformer (PET) [20] and retrain it on corn kernel data. The resulting model, CornPET, adapts to dense kernel distributions and produces interpretable point outputs.

Given point outputs, we present unicorn, a novel point-based corn row detection approach, featured by sqUeezed clusteriNg and bI-direCtional pOint seaRchiNg. These two technical improvements aim to address the challenges such as missing kernels in rows, row distortion, and row misalignment, which enables robust row detection and row-level kernel counting. Our approach offers good interpretability and adaptability to complex kernel arrangements. It is also efficient, promoting easy deployment in real-world agricultural applications.

**Fig. 1:**
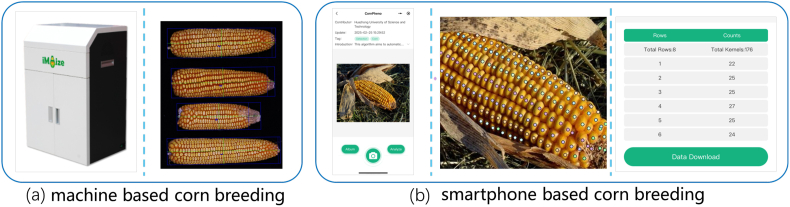
**The differences between commercial corn ear phenotyping machines and our smartphone-based corn ear phenotyping.** Commercial machines are costly and user-unfriendly, requiring corn ear transportation and controlled imaging environment. Our approach builds on a smartphone-based platform and allows for on-hand and in-situ field measurement.

To facilitate the use of CornPheno, we have deployed it on OpenPheno [21], an innovative open-access WeChat-based platform for plant phenotyping. By taking a photo, CornPheno can instantly return the number of kernels per ear, kernels per row, and rows per ear. As a free mobile platform, it avoids the costs of purchasing breeding machines and allows breeders to conduct on-site corn phenotyping, overcoming indoor machinery limitations, as shown in Fig. 1.

To evaluate our approach, we created a medium-scale dataset termed Corn Kernel Counting in the Wild (CKC-Wild), with 1, 727 images. Each image features a single corn ear from different scenarios; each kernel is manually annotated with a dot. We compare CornPheno with state-of-the-art crowd counting and plant counting approaches. The coefficient of determination (*R*^2^) for the kernels per ear and kernels per row are 0.7641 and 0.6447, respectively. CornPheno achieves the best mean absolute error (MAE) and the highest *R*^2^ values. We also provide many visualizations to intuitively demonstrate how CornPheno works. Our contributions include the following.

• CornPheno: a low-end, smartphone-based corn ear phenotyping approach capable of phenotyping kernels per ear, kernels per row, and rows per ear from a single image in complex open environment.
• unicorn: a novel point-based corn row detection approach, featured by sqUeezed clusteriNg and bI-direCtional pOint seaRchiNg, to phenotype rows per ear and kernels per row.
• CKC-Wild: a medium-scale corn kernel counting dataset with images captured from open environment, with 1, 727 images and carefully annotated point annotations.

## 2 Materials and methods

### 2.1 The CKC-wild corn kernel counting dataset

**Fig. 2:**
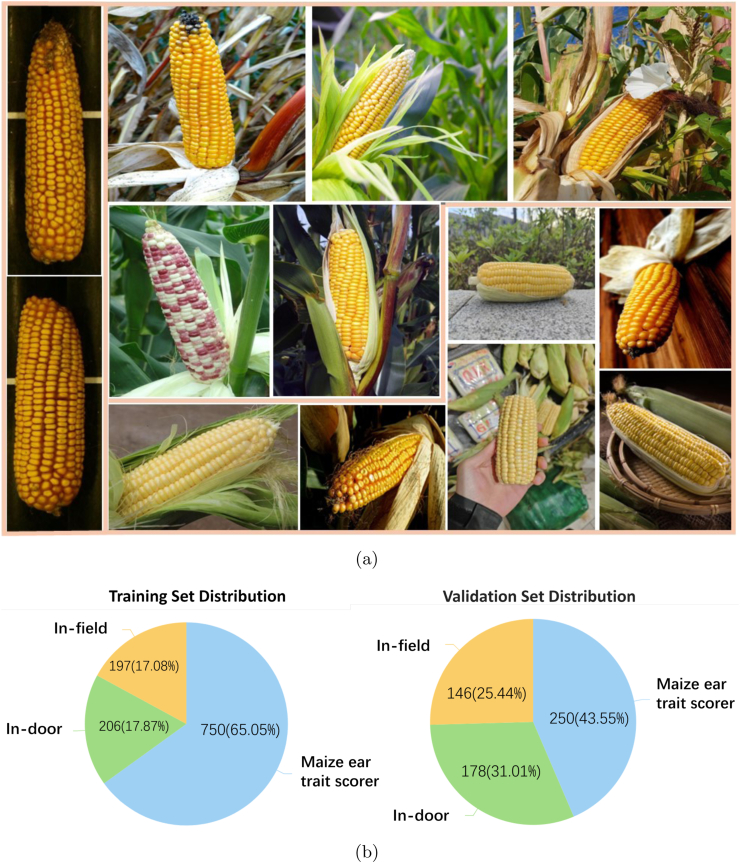
**Examples of the dataset and details of the division**. (a) Examples of the dataset; (b) Details of the data division.

The CKC-Wild corn kernel counting dataset comprises images from various sources. It includes 1, 727 images from public online data, on-site field acquisitions, and a corn ear phenotyping machine. Fig. 1 shows the corn phenotyping machine used. According to Fig. 2a, the dataset shows sufficient diversity. Images vary in size and resolution. Corn ears show differences in the number of kernels. For efficient annotation, X-AnyLabel [22] is utilized. It features tools that reduce manual effort and enhance quality. We follow strict guidelines, point-annotating each corn kernel at its geometric center. We zoom in and rotate images of occluded kernels to verify annotation accuracy. This produces a high-quality, well-annotated dataset of 1, 727 images from field, indoor, and corn phenotyping machines.

As shown in Fig. 2b, the dataset is divided into a training set (1, 153 images) and a testing set (574 images). For field environment, 197 images are used for training, and 146 for testing; for indoor environment, 206 images are used for training, and 178 for testing; for machine environment, 750 images are used for training, and 250 for testing.

### 2.2 Overview of CornPheno

**Fig. 3:**
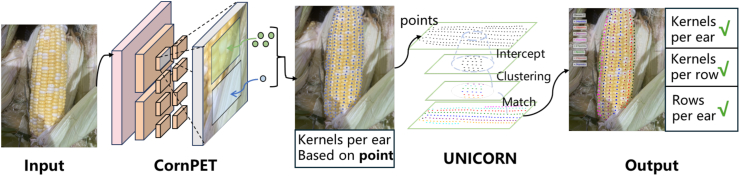
**Overview of CornPheno**. Corn ear images are input into CornPET to obtain the kernel count in the form of point coordinates, and then input the point coordinates into unicorn to derive the number of rows per ear and the number of kernels per row.

CornPheno is a straightforward approach for corn ear phenotyping, focusing on kernel counting and row detection. Fig. 3 illustrates the pipeline. The pipeline builds on CornPET, which repurposes the PET model [20], integrating efficient modules for image preprocessing, feature extraction, point querying, and transformer decoding. These modules effectively extract corn kernel positions from images. Additionally, CornPheno integrates a novel unicorn algorithm, which identifies the row structures of corn ears via squeezed clustering and bi-directional point searching. These components form a comprehensive phenotyping workflow that converts corn ear image input into phenotypic parameters.

CornPET employs point querying and transformer decoding to adapt to varying kernel densities, allowing precise and interpretable kernel localization. The unicorn row detection algorithm utilizes the localized points to infer row structures, accommodating both linear and spiral arrangements for reliable row detection. This ensures consistent row and kernel detection across different arrangements.

### 2.3 Point-query quadtree model revisited

**Fig. 4:**
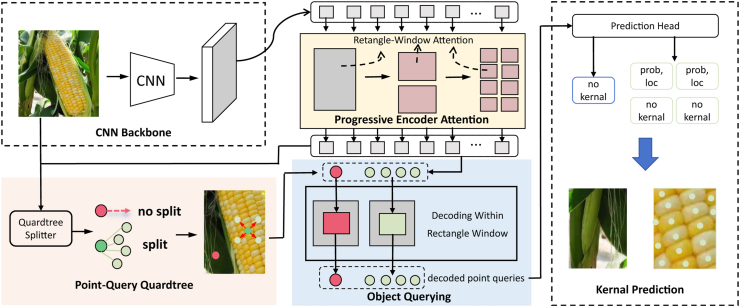
**Overall architecture of PET for corn ear kernel localization**. First, a Convolutional Neural Network (CNN) backbone is used to extract the image features. Then, a transformer encoder with progressive rectangle window attention is applied to these features to encode contextual information. Next, a quadtree splitter takes sparse querying points and the encoded features as input and outputs a point-query quadtree. After that, a transformer decoder decodes these point queries in parallel, with attention computed within a local window. Finally, these point queries are passed through a prediction head to obtain kernel predictions, i.e., whether it is “no kernel” or “kernel” along with its probability and localization.

We repurpose the PET model [20] to count corn kernels. We choose PET because its point querying mechanism provides an effective and efficient framework for counting and localizing objects. Its technical pipeline is shown in Fig. 4.

The model begins with a Convolutional Neural Network (CNN) to extract features from the input image. The encoder employs a rectangular window attention mechanism to enhance corn kernel features from global to local scales, generating the encoder feature representation ***h***_*N*_. Then the encoder feature is passed to decoder attention, which is computed by

(1) $${\hat{z}}_{l}=\text{LN}\left(\text{RectWin}-\text{SA}\left(z_{l-1}\right)+z_{l-1}\right),$$

(2) $${\hat{z}}_{l}=\text{LN}\left(\text{RectWin}-\text{CA}\left({\hat{z}}_{l},h_{N}\right)+{\hat{z}}_{l}\right),$$

(3) $$z_{l}=\text{LN}\left(\text{FFN}\left({\hat{z}}_{l}\right)+{\hat{z}}_{l}\right),$$

where ***z***_*l*−1_ is the output of the previous layer, ${\hat{z}}_{l}$ is the processed feature, and ***z***_*l*_ is the final output feature of the decoder at layer *l*. RectWin-SA and RectWin-CA are the self- and cross-attention layers, respectively. This progressive attention mechanism efficiently manages high-resolution images.

At the core of PET is its quadtree splitting design, conditioned on the encoder feature. The quadtree dynamically generates querying points from the encoder output and determines splits based on a density probability map. By downsampling the encoder feature through average pooling, the splitter uses a 1 ​× ​1 convolutional layer to extract density information and maps the density to the range of (0, 1) using a sigmoid function to generate the probability map. Higher probability values indicate denser corn kernel regions, prompting the quadtree to split querying points, thus improving awareness of dense objects. This adaptive mechanism enables accurate counting in both sparse and dense areas, enhancing flexibility in managing varying corn kernel densities.

The decoder uses cross attention to integrate quadtree features with positional information of querying points. It processes features in rectangular windows of varying sizes, generating classification probabilities and normalized pixel positions for each querying point. For each point, the classification probability *c*_*i*_ and position offset Δ*p*_*i*_ are calculated by

(4) $$c_{i}=\sigma \left(W_{c}\cdot \text{MLP}\left(z_{\text{decode}}\right)+b_{c}\right),$$

(5) $$\Delta p_{i}=W_{p}\cdot \text{MLP}\left(z_{\text{decode}}\right)+b_{p},$$

where *σ* is the sigmoid function, ***W***_*c*_ and *b*_*c*_ are classifier parameters, ***W***_*p*_ and *b*_*p*_ are position parameters, ***z***_decode_ is the decoder's output feature, and MLP is the multi-layer perceptron. The prediction head counts corn kernels by applying a threshold to the decoder output, yielding final counting and localization results.

### 2.4 CornPheno for corn ear phenotyping

This section details on how to obtain kernel-per-ear point outputs with the CornPET model and how these outputs yield kernels per row and rows per ear with our proposed unicorn algorithm. A summary of the mathematical symbols used can refer to Table S1 in the supplementary materials.

#### 2.4.1 Phenotyping kernels per ear with CornPET

CornPET maintains the identical architecture of the original PET model, only retraining the model on the CKC-Wild dataset. Note that we do not claim any technical novelty on the CornPET model. We rename PET to CornPET only to highlight its application domain. For an input corn ear image, CornPET produces a classification probability *c*_*i*_ and a normalized pixel position *p*_*i*_ = (*x*_*i*_ ​+ ​Δ*x*_*i*_, *y*_*i*_ ​+ ​Δ*y*_*i*_) for each querying point in the output stage, where (*x*_*i*_, *y*_*i*_) is the pixel location of a querying point, and (Δ*x*_*i*_, Δ*y*_*i*_) is the predicted offsets Δ*p*_*i*_. The probability *c*_*i*_ above a preset threshold is considered as a kernel. The model aggregates all point predictions to compute the total number of corn ear kernels. The output is a set of corn kernel coordinates $P=\left\{p_{i}=\left(x_{i},y_{i}\right)|1\leq i\leq n\right\}\;$, where *n* is the number of predicted points.

#### 2.4.2 UNICORN for phenotyping rows per ear and kernels per row

**Fig. 5:**
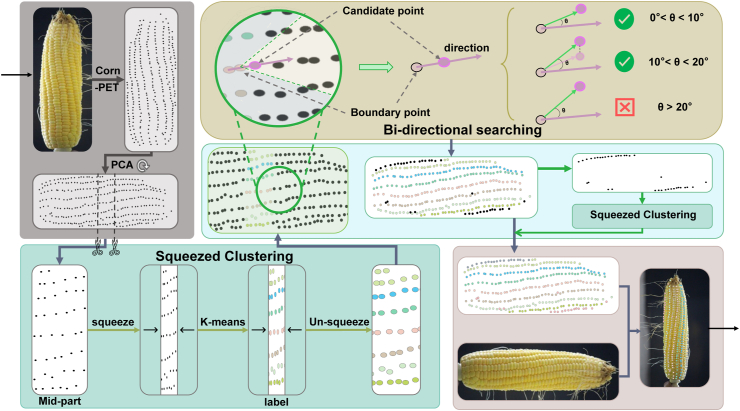
**unicorn****for detecting kernels per row and rows per ear**. We crop the central segment for K-means clustering, then use squeezed judgment vectors to select points towards both ends. An additional K-means clustering is applied to processing partial rows of the corn. Ultimately we complete the entire ear kernel detection.

Given point predictions from CornPET, it seems straightforward to directly apply standard Hough transform or RANSAC algorithms to detect rows. However, we find that they cannot robustly adapt to uneven kernel arrangements. In most cases, they even cannot return reasonable predictions. Hence, unicorn is proposed to detect corn rows. As shown in Fig. 5, it features two key designs: squeezed clustering and bi-directional point searching. We first discuss the underlying challenges of kernel arrangement.

##### 2.4.2.1 Kernel arrangement

Before detecting ear rows, a key feature is the kernel arrangement, a breeding term affecting kernel row curvature. Corn kernel arrangements can be roughly categorized into two types: spiral and linear. In a spiral arrangement, kernels are positioned along each row, spiraling with a certain angular offset and increasing towards the ends. In contrast, a linear arrangement resembles straight lines. Therefore, the linear arrangement is a special case of the spiral arrangement. To tackle these challenges, we first introduce the technique of squeezed clustering.

##### 2.4.2.2 Squeezed clustering

We first perform principal component analysis (PCA) on the model-predicted point set P to identify the principal axis and rotate it horizontally to normalize the pose of the corn ear. With P, we estimate the total length of the corn ear by *L* ​= ​*x*_max_ ​− ​*x*_min_, where *x*_max_ and *x*_min_ are the maximum and minimum *x* coordinates. To identify ear rows, a naive idea is to cluster points by rows. Instead of treating all points equally, we choose a reliable subset of points from the middle segment of the corn ear, because we observe that this segment typically exhibits a quasi-grid arrangement, which simplifies row clustering. After further applying horizontal compression on these chosen points, corn rows can be effectively aggregated. Then, the aggregated kernel coordinates can be easily clustered using the K-means clustering algorithm. To define the longitudinal span of the sampled segment, we introduce a hyperparameter *α*. It controls the number of points sampled by an interval of *α* ​× ​*L*. The center of the interval is also the center of the corn ear (*L*/2). These sampled points form the set of middle-segment points, denoted by $P_{m}$. The horizontal compression is controlled by a hyperparameter *t*, which squeezes the *x* coordinates by a factor of *t* such that $P_{m}$ is mapped to

(6) $$P_{m}^{\prime }=\left\{p_{i}^{\prime }=\left(\frac{x_{i}}{t},y_{i}\right)|\left(x_{i},y_{i}\right)\in P_{m}\right\}.$$

To determine the optimal number of clusters in K-means clustering, the silhouette coefficient is employed to evaluate the quality of clustering. To search for the optimal row count *C*, we assess *C* in the range of 2 ​≤ ​*C* ​≤ ​10 for unilateral measurement, leveraging a biological prior that the number of the whole corn ear rows often ranges from 12 to 20.

In particular, we compute the silhouette coefficient *s*(*i*) for the *i*-th point in $P_{m}^{\prime }$ by

(7) $$s\left(i\right)=\frac{b\left(i\right)-a\left(i\right)}{\max\left(a\left(i\right),b\left(i\right)\right)},$$

where

(8) $$a\left(i\right)=\frac{1}{|P_{m,k}^{\prime }|-1}\underset{j\in P_{m,k}^{\prime },j\neq i}{\sum }d\left(i,j\right),$$

and

(9) $$b\left(i\right)=\min_{k\neq i}\frac{1}{\left|P_{m,k}^{\prime }\right|}\underset{j\in P_{m,k}^{\prime }}{\sum }d\left(i,j\right).$$

*a*(*i*) is the average distance of ***p***_*i*_ from all other points within the same cluster, and *b*(*i*) is the average distance of ***p***_*i*_ from points in other clusters. $P_{m,k}^{\prime }$ refers to the point set of the cluster *k*, $\left|P_{m,k}^{\prime }\right|$ represents the number of data points in the cluster *k*, and *d*(*i*, *j*) indicates the *ℓ*_2_ distance between the *i*-th and *j*-th points.

We compute the average silhouette coefficient for the *c*-class clustering by $S\left(c\right)=\frac{1}{n}\sum_{i=1}^{n}s\left(i,c\right)$, where *s*(*i*, *c*) is the silhouette coefficient when *C* ​= ​*c*. We select the optimal number of clusters *C* ​= ​*c*∗ based on the largest average silhouette coefficient as

(10) $$c^{*}=\underset{c}{\arg\;\max}S\left(c\right).$$

Finally, we assign the cluster label to each point in $P_{m}^{\prime }$ and transfer the label from $P_{m}^{\prime }$ to P.

##### 2.4.2.3 Bi-directional point searching

Using the row-clustered points above, we can extend them from the center to both ends through a bi-directional search, effectively identifying entire rows. We first split P into two parts: $P_{l}$, which includes points with labels in P, and $P_{u}$, which contains points without labels. For each class in $P_{l}$, treated as a row of corn, we identify the leftmost and rightmost points as boundary points $P_{b}=\left\{p_{b}^{k}|1\leq k\leq C\right\}$, where *k* refers to the *k*-th row, and $p_{b}^{k}$ denotes the leftmost point when searching left or the rightmost point when searching right. Each $p_{b}^{k}$ is assigned a unit vector pointing right if it is the rightmost point, and the opposite if it is the leftmost. These unit vectors form the vector set $V=\left\{v^{k}|1\leq k\leq C\right\}$, where ***v***^*k*^ is the vector of *k*-th row. When searching to one end, we select the point ***p***_new_ closest to the center of that end in $P_{u}$ as our candidate point to obtain the candidate vectors $V_{\text{new}}=\left\{v_{\text{new}}^{k}=\vec{p_{b}^{k}p_{\text{new}}}|1\leq k\leq C\right\}$. We calculate the angle *θ*_*k*_ between ***v***^*k*^ and $v_{\text{new}}^{k}$ by

(11) $$\theta_{k}=\cos^{-1}\left(\frac{v^{k}\cdot v_{\text{new}}^{k}}{\left|v^{k}||v_{\text{new}}^{k}\right|}\right).$$

We use *θ*_*k*_ to guide point searching. If *θ*_*k*_ meets certain criteria, we label the point ***p***_new_, substituting $p_{b}^{k}$ for ***p***_new_ and ***v***^*k*^ for $v_{\text{new}}^{k}$. This makes ***p***_new_ the new boundary point $p_{b}^{k}$, completing the first iteration. We then select a new candidate point and iterate for all points in $P_{u}$.

##### 2.4.2.4 Position correction in bi-directional point searching

Since $P_{u}$ does not align perfectly with the center of corn kernels during each iteration, a candidate point ***p***_new_ may form a rough curve. The positional fluctuation of ***p***_new_ can lead to an unreliable vector set V. For points requiring angular correction, we keep their x-coordinates fixed and only adjust their y-coordinates to achieve the desired angle. After completing the positional modification, the points are repositioned back to their original locations once their labels have been assigned. To address this, we use the angle *θ*_*k*_ between $v_{\text{new}}^{k}$ and ***v***^*k*^ to correct points with an angle threshold *θ*_*T*_:

a) 0 < *θ*_*k*_ ≤ *θ*_*T*_. Angles within this range suggest corn ear rows are of low angular divergence and conform to the normal morphological curvature; no correction is required.
b) *θ*_*T*_ < *θ*_*k*_ ​≤ ​2*θ*_*T*_. Angles within this range imply angular deviations are mainly affected by model prediction errors, particularly for marginal kernel positions. When predicted coordinates exhibit spatial inconsistency relative to ground-truth kernel centroids, fluctuations can propagate along with the natural curvature of the corn ear. To mitigate this artifact, we move the *y* coordinate only such that *θ*_*k*_ is reduced to 0.5*θ*_*k*_. This aligns with our empirical observation that ground-truth inter-row angles distribute mainly between 5° and 10° (Fig. 8).
c) *θ*_*k*_ ​> ​2*θ*_*T*_. When the angular deviation exceeds 2*θ*_*T*_, point correction is also disabled due to the risk of catastrophic error propagation in row tracing. Such points would be treated as outliers.

The rationale behind this rule-based design will be validated via parameter sensitivity analysis in Section 3.

##### 2.4.2.5 Partial row processing by clustering twice

After correcting the positions in bidirectional point searching, most row detections are complete. However, the spiral arrangement of corn kernels results in partial rows that appear at both ends. Rows from the center and front shift to the back and become hidden toward the end, while those at the back become invisible. Consequently, two partial rows are often observed at both corn ear boundaries. We address this by applying the squeezed clustering algorithm twice on the unlabeled point set, wherein the number of clusters *C* is only evaluated within the interval from 1 to 2. Finally, additional labels are assigned to the unlabeled points based on the clustering results, and the newly labeled points are combined with the previously labeled set to generate the final row detection result. The unicorn algorithm is summarized in Algorithm 1.

Algorithm 1 Unicorn

**Table:** 

**Input:** CornPET generated point set P
1: Squeezing factor *t*
2: Base angle threshold *θ*_*T*_
3: Segment sampling ratio *α*
**Output:** Labeled row point set $L\subseteq \left\{\left(p_{i},k_{i}\right)\right\}$
4: P←PCA(P)
5: $P^{\prime }\leftarrow$ extract the middle 2*α* segment points of P
6: $P_{m}^{\prime }\leftarrow \left\{\left(\frac{x}{t},y\right)|\left(x,y\right)\in P_{m}\right\}$ ⊳ squeeze *x* coordinates per Eq. ​(6)
7: $c^{*}\leftarrow s^{*}≜\underset{c}{\mathrm{argmax}}S\left(c\right)$ ⊳ find the optimal cluster configuration per Eq. ​(10)
8: $L^{\prime }\leftarrow \text{K}-\text{Means}\left(P_{m}^{\prime },c^{*}\right)$ ⊳ initial clustered point set, $L^{\prime }\subseteq \left\{\left(\left(x_{i}^{\prime },y_{i}\right),k_{i}\right)\right\}$
9: $L\leftarrow \left\{\left(\left(t\times x^{\prime },y\right),k\right)|\left(\left(x^{\prime },y\right),k\right)\in P_{l}^{\prime }\right\}$
10: $P_{l}\leftarrow \left\{p^{k}|\left(p^{k},k\right)\in L\right\}$
11: $P_{u}\leftarrow P\setminus P_{l}$
12: $L_{b}\leftarrow \text{GetBoundaryPoints}\left(L\right)$ ⊳ get initial boundary points, $L_{b}\subseteq L$
13: **for all** ​***p***_*u*_ in $P_{u}$ ​**do**
14: **for all** ​(***p***_*b*_, k) in $L_{b}$ ​**do**
15: ***v***_new_ ←***p***_new_ ​− ​***p***_*b*_
16: $\theta_{k}\leftarrow \arccos\left(\frac{v\cdot v_{\text{new}}}{\left|v\|v_{\text{new}}\right|}\right)$ ⊳ compute angle per Eq. ​(11)
17: **if** ​*θ*_*k*_ ​< ​*θ*_*T*_ ​**then** ⊳ position correction per a)
18: $L\leftarrow L\cup \left\{\left(p_{\text{new}},k\right)\right\}$
19: $P_{l}\leftarrow P_{l}\cup \left\{p_{\text{new}}\right\}$
20: $P_{u}\leftarrow P_{u}\setminus \left\{p_{\text{new}}\right\}$
21: ***p***_*b*_ ←***p***_new_
22: ***v*** ←***v***_new_
23: **break**
24: **else** ​**if** ​*θ*_*T*_ ​≤ ​*θ*_*k*_ ​< ​2*θ*_*T*_ ​**then** ⊳ position correction per b)
25: $L\leftarrow L\cup \left\{\left(p_{\text{new}},k\right)\right\}$
26: $P_{l}\leftarrow P_{l}\cup \left\{p_{\text{new}}\right\}$
27: $P_{u}\leftarrow P_{u}\setminus \left\{p_{\text{new}}\right\}$
28: ***p***_*b*_ ←***p***_new_
29: $v\leftarrow \left(v_{\text{new}}^{x},\tan\left(0.5\times \theta_{k}\right)v^{x}\right)$
30: **break**
31: **end** ​**if**
32: **end** ​**for**
33: **end** ​**for**
34: $P_{u}^{\prime }\leftarrow \left\{\left(\frac{x}{t},y\right)|\left(x,y\right)\in P_{u}\right\}$
35: $c_{\text{half}}^{*}\leftarrow \underset{c_{\text{half}}\in \left\{1,2\right\}}{\mathrm{argmax}}S\left(c;P_{\text{u}}^{\prime }\right)$ ⊳ find the optimal cluster configuration per Eq. ​(10)
36: **if** ​$s_{\text{half}}^{*}≜S\left(c_{\text{half}}^{*};P_{\text{u}}^{\prime }\right)>-1$ ​**then**
37: $P_{\text{half}}\leftarrow \text{K}-\text{Means}\left(L_{\mathrm{half}}^{\prime },c_{\text{half}}^{*}\right)$
38: $L\leftarrow L\cup L_{\text{half}}$
39: **end** ​**if**

### 2.5 Accessibility on WeChat mini-program

**Fig. 6:**
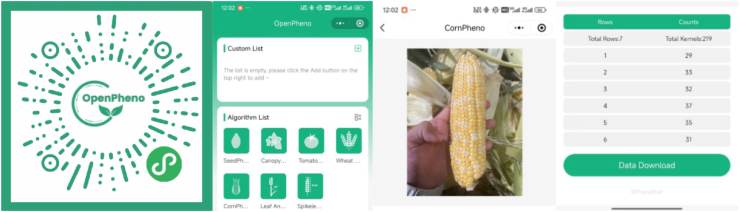
**The Mini-Program interface and a use case**.

CornPheno targets open environments including the field. To enhance the accessibility of CornPheno and ensure ease of use for breeders, we have integrated the algorithm into a WeChat-based Mini-Program platform OpenPheno [21], as illustrated in Fig. 6. WeChat is the world's most widely used social media, and OpenPheno is an open-access, smartphone-based platform for instant phenotyping at hand, enabling users to conduct plant phenotyping for free. Within OpenPheno, users can select the CornPheno tool, capture and upload an image, and click “Analyze” to initiate the phenotypic data extraction process. The returned data includes the processed image along with key phenotypic traits such as kernels per ear, kernels per row, and rows per ear. Model inference is performed on the OpenPheno server, and no on-device computation is carried out. The average inference time on the server is 1.86 ​s for CornPET and 0.11 ​s for UNICORN. This server-side deployment ensures responsive performance while minimizing the computational burden on mobile devices. In this way, users can use our approach using their smartphones anytime and anywhere.

### 2.6 Implementation details

#### 2.6.1 Data augmentation and training details

To enhance training, we first augment the training images by image scaling and cropping. In particular, we randomly crop image patches of size 256 ​× ​256 from each scale of image pyramid. To enhance the robustness of CornPheno in outdoor environment, we additionally augment image patches through random scaling, rotation, and flipping.

We set the initial learning rate of 1*e*^−5^ for the CNN backbone and 1*e*^−4^ for the rest of the model. The model was trained for 700 epochs. The rest experimental configurations were kept the same as the original PET model.

#### 2.6.2 Parameter setting in UNICORN

unicorn involves three key hyperparameters: the compression coefficient *t*, the segment sampling ratio *α*, and the angular threshold *θ*_*T*_. To evaluate the rationality of these parameter configurations and assess their sensitivity, a systematic ablation study was conducted. Due to the spatially and functionally distinct roles of the parameters, we assume the relative independence between (*t*, *α*) and *θ*_*T*_ and evaluate them respectively.

• *Evaluation subset and protocol*: A fixed subset set of 50 high-resolution images of individual corn ears with clear kernel visibility was chosen. These images were processed through the CornPET model to generate kernel position predictions.
• *Evaluation metric*: Given that the core objective of *t* and *α* is to optimize row-wise kernel clustering performance for ear phenotyping, the silhouette coefficient averaged over the 50 samples was adopted as the metric. Values approaching 1 indicate superior intra-cluster compactness and inter-cluster separation (i.e., more distinct row boundaries), signifying enhanced row-count prediction accuracy. The mean silhouette coefficient is defined by

$$\bar{S}=\frac{1}{N}\overset{N}{\underset{i=1}{\sum }}S_{i},$$

where *S*_*i*_ is the silhouette coefficient of the *i*-th sample, and *N* is the total number of samples.

a) *t* and *α*: Both parameters (*t*—compression coefficient; *α*—segment sampling ratio) fundamentally govern subsequent clustering performance. Since the silhouette coefficient evaluates the inter-sample distance, we first analyze the working mechanism of *t*.

Mathematically, when *t* ​≥ ​100, the contribution of *x*-coordinate to Euclidean distance decays quadratically, characterized by the scaling factor:

$$w_{x}=\frac{1}{t^{2}},$$

where *w*_*x*_ denotes the *x*-coordinate weight in the distance metric. This implies that, when *t* is beyond a threshold, the variance of *x*-coordinate becomes negligible in distance computations such that $\lim_{t\to \infty }\mathrm{Var}\left(x\right)\to 0\;\text{in}\;d\left(p_{i},p_{j}\right)=\sqrt{w_{x}\Delta x^{2}+\Delta y^{2}}$. Consequently, further increasing *t* exponentially can diminish the influence of *x* component in the distance metric and the partial derivative of silhouette coefficient

(12) $$\frac{\partial }{\partial t}\left(\frac{\partial d}{\partial \Delta x}\right)=-2t^{-3}\Delta x,\;t>100.$$

**Fig. 7:**
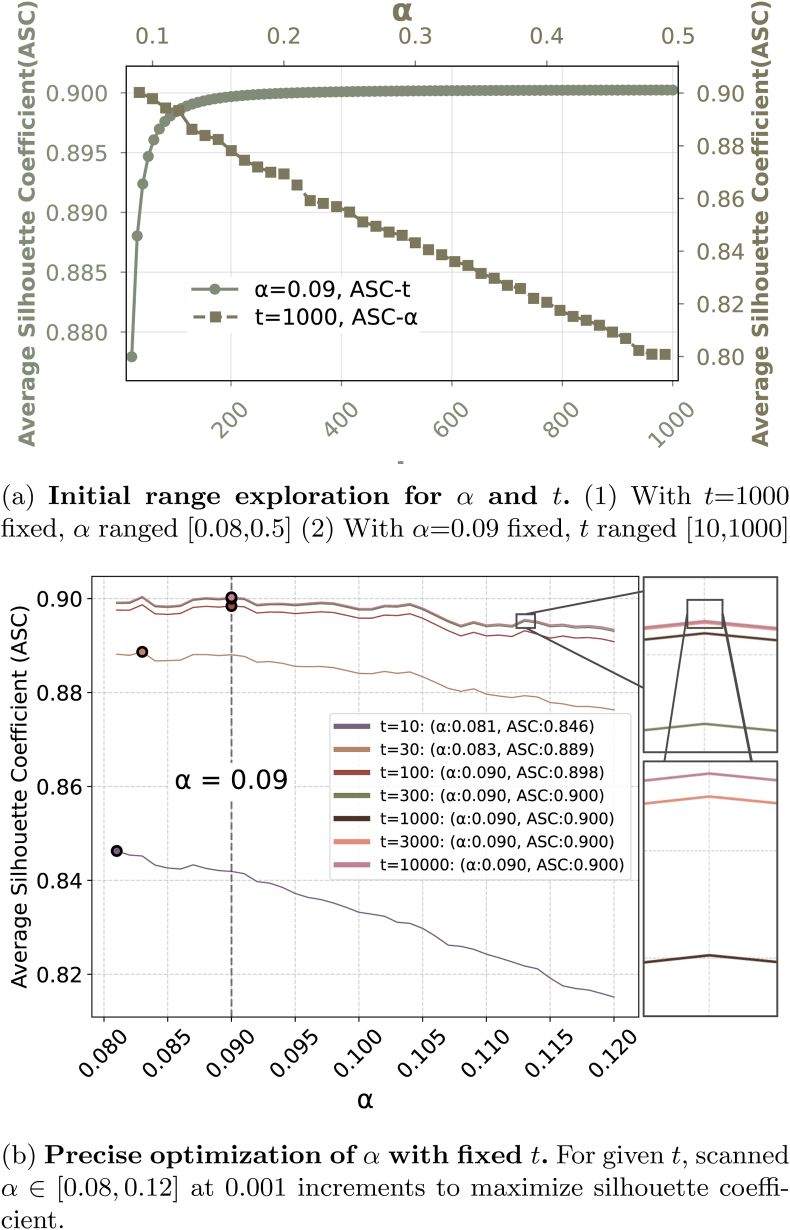
**Parameter Optimization for*t*and*α***. (1) shows the impact of *α* and *t* on clustering effectiveness and identifies their optimal ranges. (2) demonstrates refined values for these parameters.

Eq. (12) also confirms the exponential decay of the *x* component with increased *t*. To pre-investigate the optimal value ranges for *t* and *α*, we initially fixed *t* at 1000. This sufficiently large value ensures negligible dimensional influence from *t* when *α* varies. Then, we evaluate *α* across the biologically relevant interval [0.01, 0.50] using a step size of 0.01, while computing the corresponding mean silhouette coefficient. Our analysis in Fig. 7a reveal that, when *α* ​∈ ​[0.01, 0.07], the limited segment sampling yield insufficient point density for reliable K-means clustering. When *α* ​∈ ​[0.08, 0.50], the mean silhouette coefficient exhibits monotonic decreasing when *α* ​> ​0.09. This decline indicates deteriorated clustering quality and decreased row detection accuracy with increased sampling spans. Subsequently, we constrain the segment sampling ratio to *α* ​∈ ​[0.08, 0.12] with a finer step size of 0.001. Concurrently, seven logarithmically spaced values of the compression coefficient (*t* ​= ​10, 30, 100, 300, 1000, 3000, 10000) are evaluated as well.

**Fig. 8:**
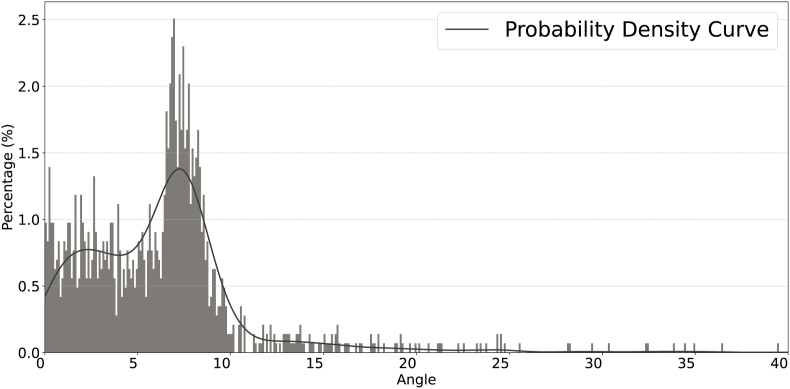
**Angle distribution with Probability Density Curve**. We conducted a statistical analysis of the angle values ranging from 0° to 40° and performed a curve fitting on the probability of occurrence for each angle value.

For each *t*, we analyze the relationship between *α* and $\bar{S}$. Results are shown in Fig. 7b. One can observe that when *t* ​≥ ​300, $\bar{S}$ approaches the theoretical maximum limit. Crucially, when *t* ​≥ ​1000 (tested at *t* ​= ​1000, 3000, 10000), the response curves of $\bar{S}$ versus *α* become indistinguishable. This convergence indicates negligible improvements in the clustering quality beyond the compression threshold, that is, *t* ​≈ ​1000. According to the study of parameter sensitivity, we choose the compression coefficient *t* ​= ​1000 and segment sampling ratio *α* ​= ​0.09 as the default hyperparameters.

b) *θ*_*T*_: To find biological evidence for the choose of the angular threshold *θ*_*T*_, we perform a statistical analysis using the 50 corn ear images. In particular, 48 valid rows are manually annotated to identify morphologically intact kernel rows. For each row, inter-kernel vector $\vec{P_{1}P_{2}}$ and $\vec{P_{2}P_{3}}$ are constructed among consecutive triplets of kernels (*P*_1_, *P*_2_, *P*_3_), with the inter-vector angle *θ* computed as
  (13) $$\theta =\cos^{-1}\left(\frac{\vec{P_{1}P_{2}}\cdot \vec{P_{2}P_{3}}}{\left\|\vec{P_{1}P_{2}}\|\|\vec{P_{2}P_{3}}\right\|}\right)$$

The angular distribution from 1, 441 measurements is illustrated in Fig. 8 and Table 1. It shows that 91.3 ​% angles are less than 10°, revealing a natural constraint of corn ear growth. Quantitative analysis of the angular distribution identifies that *θ*_*T*_ ​= ​10° is a statistically significant point.

**Table 1:** Statistical distribution of inter-kernel angles. The majority is in **boldface**.

Angular range	Count	Percentage (%)
*θ* ​> ​40	6	0.416
30 ​< ​*θ* ​≤ ​40	8	0.555
20 ​< ​*θ* ​≤ ​30	19	1.319
10 ​< ​*θ* ​≤ ​20	**93**	**6.454**
**0 ​≤ ​*θ* ​≤ ​10**	**1315**	**91.256**

## 3 Results

Here we begin with comparing baselines and the evaluation metrics employed. Then, we report the results of counting kernels per ear, rows per ear, and kernels per row.

### 3.1 Baselines and evaluation metrics

#### 3.1.1 Baselines

To validate the effectiveness of CornPheno, we compare it with five well-established plant counting models, including BCNet [23], CSRNet [24], P2PNet [25], TasselNetV2 [26], TasselNetV3-Lite [27], and CCTrans [28] as follows:

• **BCNet**: BCNet employs a blockwise classification idea to address sample imbalance;
• **CSRNet**: CSRNet combines a CNN front-end for feature extraction and a dilated CNN back-end to enable accurate density estimation;
• **P2PNet**: P2PNet is a point-to-point framework for dense crowd localization. It directly predicts object coordinates, avoiding standard bounding boxes. By combining classification and regression losses, it achieves high accuracy in overlapping and occluded scenes;
• **TasselNetV2**: TasselNetV2 is a CNN-based model for corn tassel counting, using local regression to predict local counts from images. Robust to occlusion, lighting variations, and dense distribution, it serves as a reliable baseline for high-throughput plant counting;
• **TasselNetV3-Lite**: TasselNetV3-Lite is an extended version of TasselNetV2. Optimized for field use, it ensures real-time performance on low-resource devices, achieving high accuracy in cluttered environments.
• **CCTrans**: CCTrans is a transformer-based model for crowd counting, leveraging pyramid vision transformers to aggregate multi-scale contextual features. Robust to perspective distortion, scale variations, and dense crowds, it establishes a strong baseline for accurate and efficient high-throughput people counting.

#### 3.1.2 Evaluation metrics

We use the Mean Absolute Error (MAE), Mean Squared Error (MSE), and the coefficient of determination *R*^2^ to evaluate the performance of kernels per ear. They take the form

(14) $$MAE=\frac{1}{N}\overset{N}{\underset{i=1}{\sum }}\left|C_{i}^{g}-C_{i}^{p}\right|,$$

(15) $$MSE=\frac{1}{N}\overset{N}{\underset{i=1}{\sum }}{\left(C_{i}^{g}-C_{i}^{p}\right)}^{2},$$

(16) $$R^{2}=1-\frac{\sum_{i=1}^{N}{\left(C_{i}^{g}-C_{i}^{p}\right)}^{2}}{\sum_{i=1}^{N}{\left(\bar{C_{i}^{g}}-C_{i}^{p}\right)}^{2}},$$

where *N* is the number of images, $C_{i}^{g}$ and $C_{i}^{p}$ are ground-truth image count and predicted image count of the *i*th image, respectively, and $\bar{C_{i}^{g}}$ is the mean ground-truth count.

For evaluating rows per ear, aside from the MAE, MSE, and *R*^2^ metrics, and the zero-error rate is also used as the evaluation metric, where the zero-error rate indicates the percentage of correct predictions.

For evaluating kernels per row, we also consider other metrics such as Precision, Recall, and *F*_1_-Score, because large angles (>20°) can lead to erroneous row detections such that some kernels are assigned to nearby rows. In this case, it is suitable to be treated as a classification task. Precision, Recall, and *F*_1_-Score are defined by

(17) $$\text{Precision}=\frac{\text{TP}}{\text{TP}+\text{FP}},$$

(18) $$\text{Recall}=\frac{\text{TP}}{\text{TP}+\text{FN}},$$

(19) $$F_{1}-\text{Score}=2\times \frac{\text{Precision}\times \text{Recall}}{\text{Precision}+\text{Recall}},$$

where TP is the True Positive indicating correct kernel assignment, FP is the False Positive referring to kernels assigned to an incorrect row, and FN is the False Negative representing valid kernels that are undetected in their correct rows.

### 3.2 Evaluation of kernels per ear

**Fig. 9:**
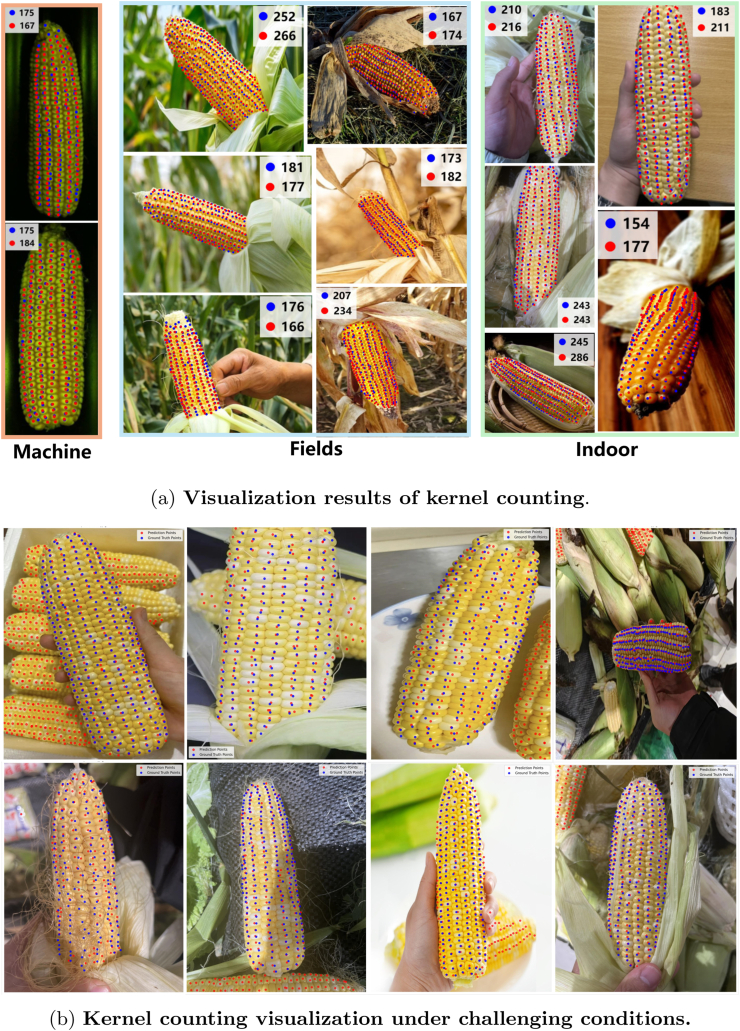
**Visualization of kernel counting in various scenarios.** The blue points are the labeled points, and the red points are predicted ones.

Quantitative and qualitative results are shown in Table 2 and Fig. 9a, respectively. According to Table 2, our model outperforms other competitors, especially in terms of the *R*^2^ metric. Compared with previous approaches, CornPET leads to a remarkable improvement in *R*^2^, demonstrating a 0.09 ∼ 0.18 gain over the baseline model. CornPET reaches an *R*^2^ value of 0.7641, significantly outperforming other approaches such as P2PNet (*R*^2^ ​= ​0.6884), TasselNetV2 (*R*^2^ ​= ​0.6833), and CCTrans (*R*^2^ ​= ​0.6897). Visualizations of three different scenes in Fig. 9a suggest that CornPET is robust and has good interpretability. To further analyze the performance under different scenarios, the test set was divided into a machine subset, an indoor subset, and a field subset. The results shown in Table 3 reveal that the controlled machine environment reports the best performance (*R*^2^ ​= ​0.8322), and the field performance is slightly lower than that in the machine-captured background but is still competitive (*R*^2^ ​= ​0.8190). This means our algorithm can maintain good robustness in complex scenarios. For the indoor condition, the results are unsatisfactory (*R*^2^ ​= ​0.5600). We find that there exist multiple corn ears in the background, but only one ear is annotated (Fig. 9b), which introduces significant over-estimations. After removing those images with multiple corn ears, the performance improves (*R*^2^ ​= ​0.7498).

CornPET achieves the highest *R*^2^ value (0.7641) among all methods, confirming its superiority. Its MSE metric (34.82) is slightly higher than TasselNetV2 (33.71) and P2PNet (33.99), despite reporting the lowest MAE (22.06). This suggests the over-estimation in indoor environment affects the variance. To address this limitation, instance segmentation may be used to isolate individual corn ears before kernel counting, but this may be beyond the scope of current work.

### 3.3 Evaluation of rows per ear

**Fig. 10:**
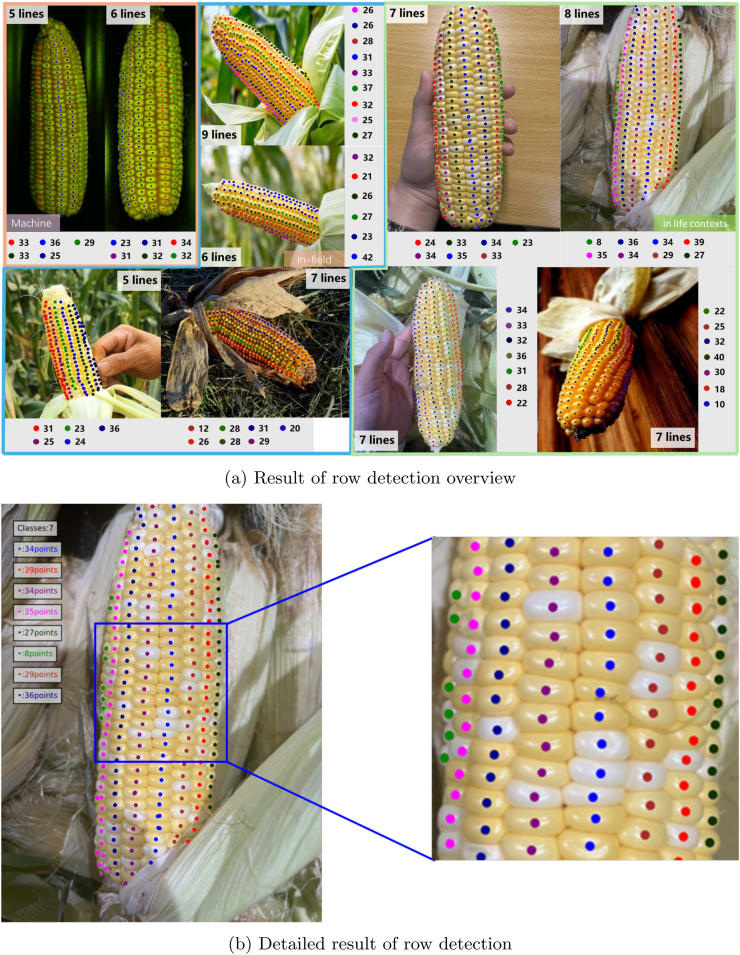
**Visualization of row detection in various scenarios**. (a) Presents an overview of row detection performance in different environments. (b) Provides detailed examples of rows per ear and kernels per row for specific samples.

**Fig. 11:**
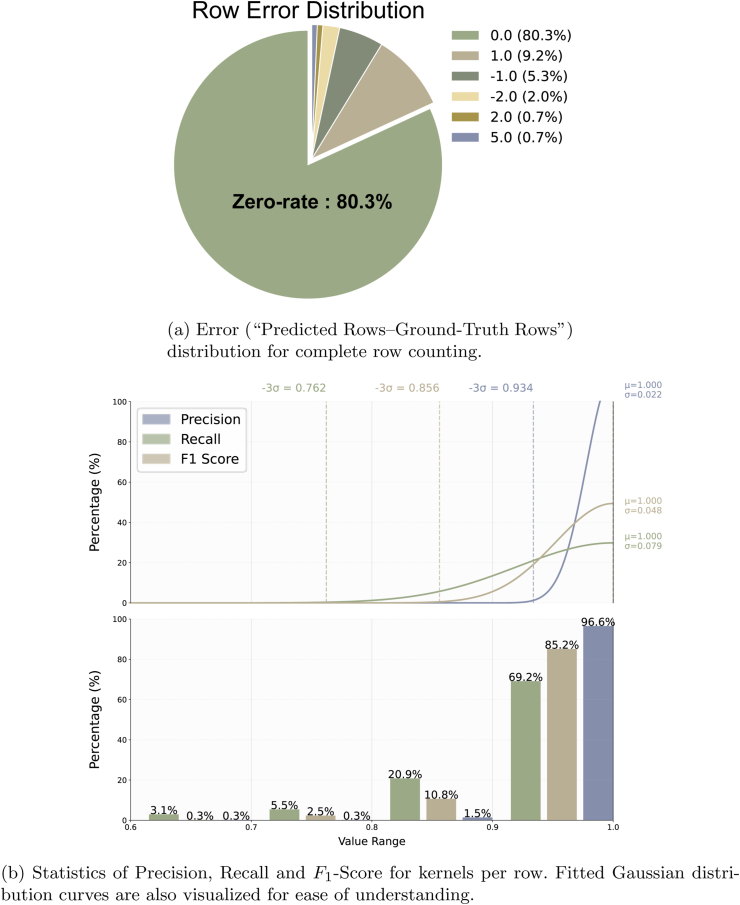
Evaluation of row detection and kernels per row.

After obtaining the kernel count results, we utilize point coordinates and image metadata for corn row detection. Our approach effectively detects complete rows in three scenarios Fig. 10a. Qualitative results are visualized in Fig. 10b We selected 50 images for each of the three scenarios in the test set, obtained 150 samples each and estimated the number of rows. In Fig. 11a, the largest percentage of rows detected is 0, i.e., the number of rows is predicted correctly, and the percentage reaches 80.3 ​%. The MAE in number of rows reaches 0.234 rows per ear, and the zero-rate reaches 80.3 ​%. The portion of the majority of the rows is 10.4 ​% and the portion of the minority is 7.3 ​%. Both are rather close compared to the zero error rate.

### 3.4 Evaluation of kernels per row

Kernels per row refer to the true kernel count of each physical row on a single corn ear (not the average value). unicorn generates per-row kernel predictions, and then manual annotation provides corresponding row-matched ground truths. Experimental results (Fig. 11) are derived from direct comparison of verified row pairs following manual correspondence validation.

To avoid ambiguities of partially observable ear rows (“partial rows”) and annotation artifacts, we manually inspect row-count and kernel-per-row statistics across the 50 benchmark images. Since the definition of “partial rows” is vague and subjective, we only compare rows per ear and kernels per row in complete rows. For an accurate evaluation, we manually selected all rows from the 50 benchmark images that were clearly identifiable by eye and appeared largely complete from tip to base to serve as the ground truth for evaluation. In Table 4, the MAE for kernels per row across these 50 images reaches 2.43, the MSE reaches 11.84, and the *R*^2^ reaches 0.6447. In Fig. 11b, the distribution of *F*_1_-Score, Precision and Recall bounded in [0, 1] are analyzed by constructing a decile-binned (Δ ​= ​0.1) frequency histogram, and Gaussian curves are also fitted. Most of the three metrics are distributed between 0.9 and 1.0. The *F*_1_-Score is 0.9418, the Precision is 0.9796, and the Recall is 0.9091. Based on the results of the curve fitting, we can also learn that the −3*σ* of the three metrics are sufficiently large, which means they can be ignored outside −3*σ*. These data reflect the confidence interval of unicorn.

**Table 4:** Performance of MAE, MSE, and *R*^2^ of kernels per row and rows per ear.

	MAE	MSE	*R*^2^
Rows per ear	0.23	0.42	0.7041
Kernels per row	2.43	11.84	0.6447

## 4 Discussions

### 4.1 CornPheno indicates an effective and interpretable solution for corn ear phenotyping

Table 2 indicates that CornPET significantly outperforms other models in terms of applicability to field environments. One can see that the predicted points are rather close to the ground-truth points in terms of both quantity and localization, as illustrated in Fig. 9a. unicorn also shows reliable results, as shown in Fig. 11. Fig. 10 further illustrates its capability to extract rows per ear and kernels per row across various ear shapes, row arrangements, and environmental conditions. We also note that, the MSE of CornPET is slightly higher than other models despite having the lowest MAE. Our analysis reveals that this is due to background interference from multi-ear images in indoor settings (Table 3). This could be addressed in future work with pre-processing steps like object detection or instance segmentation.

**Table 2:** Performance of MAE, MSE, and *R*^2^ of different approaches. Best performance is in **boldface**.

Method	#Param (M)	MAE	RMSE	*R*^2^
BCNet	14.8	22.27	36.96	0.6065
CSRNet	16.3	24.62	37.97	0.5832
P2PNet	21.6	23.58	33.99	0.6884
TasselNetV2	16.0	23.08	**33.71**	0.6733
TasselNetV3-Lite	0.3	25.74	40.91	0.5860
CCTrans	27.33	22.68	33.84	0.6897
CornPET (Ours)	20.9	**22.06**	34.82	**0.7641**

**Table 3:** Performance of MAE, MSE, and *R*^2^ under different imaging conditions. Indoor∗ means the results excluding disturbed corn ears in the background.

Conditions	MAE	MSE	*R*^2^
Machine	27.78	32.35	0.8322
Indoor	22.36	38.71	0.5600
Indoor∗	16.88	25.46	0.7498
Field	20.04	30.20	0.8190

All these results render CornPheno a convincing solution: CornPheno not only enables the acquisition of kernels per ear, rows per ear, and kernels per row directly on a smartphone via in-field photography, but also demonstrates robustness and interpretability in complex field environments, making it a potential tool for in-situ measurements.

### 4.2 The output form of CornPET significantly enhances the transparency and credibility of phenotyping

CornPET adopts a point-query mechanism that directly outputs the coordinates of each corn kernel. This approach provides a clear and precise representation of kernel locations. The explicit localization is highly interpretable, as it allows for straightforward verification of the counting results and serves as a solid foundation for subsequent analyses. The detailed point predictions generated by CornPET are particularly useful for coordinate-based row detection by unicorn, which relies on these precise coordinates to accurately identify and analyze the row structures of corn ears. The high interpretability of CornPET is a key feature that enhances the overall transparency and reliability of our phenotyping workflow.

Compared with conventional bounding-box object detection, our point-querying paradigm offers additional benefits such as annotation efficiency. On average, a single image can be annotated within one to 2 ​min using points, which is only 40 percent of the time used by bounding boxes (four to 5 ​min), yielding a significant reduction in manual effort. Compared with density-based approaches, point querying outputs the object center, eliminating hyper-parameter tuning in density-map estimation, thereby improving interpretability. Moreover, under challenging conditions – overlapping leaves, dense plant populations, and partial occlusions – the single-click label remains unique and stable, effectively suppressing false positives and false negatives, which improves the robustness of the method.

### 4.3 Analysis of row count underestimation

In counting rows per ear, there is basically no underestimation or overestimation that for the detection of the number of complete rows from Section 3.3. However, for all rows in corn ears, the definition of *partial rows* remains inherently ambiguous. Our algorithm categorizes any incomplete kernel row as a partial row, resulting in significant morphological heterogeneity under field conditions. These span near-complete rows with minor kernel omissions to severely fragmented rows exhibiting almost unobservable kernels.

During final quantification, all detected partial rows are counted as **full rows**. However, during row structure identification, *severely fragmented partial rows* pose substantial recognition challenges. Such rows exhibit high susceptibility to erroneous merging with adjacent intact rows during clustering or curve-fitting procedures. This merging propensity directly contributes to underestimation (i.e., omission of valid rows), a systemic error that is difficult to eliminate within the current algorithmic framework.

In addition to the partially observable rows, occlusions (i.e., mutual obstruction among kernels) further deteriorate partial row recognition. For severely fragmented rows, even observable kernels often cannot be accurately localized in single-view images. This incomplete detection not only diminishes the row's intrinsic visibility but also impedes algorithmic discrimination of fragmented rows as independent structural entities.

To address these challenges, further studies may be required from three aspects: i) *quantitative partial row definition*: rigorous, measurable criteria should be established for partial row classification, for instance, based on observable kernel proportion ≥50 ​% or continuity index thresholds; ii) *advanced row identification algorithms*: optimized clustering/curve-fitting row detection algorithms should be developed with enhanced robustness to fragmented structures, specifically improving differentiation between adjacent intact rows and partial rows; iii) *high-performance kernel detection models:* advanced kernel detection frameworks (e.g., state-of-the-art instance segmentation networks) should be developed to improve localization accuracy for sparse kernels, thereby providing salient features for fragmented partial rows.

### 4.4 UNICORN indicates a robust way to detect corn ear rows

Fig. 10 demonstrates the advanced capabilities of our row detection algorithm, highlighting four key advantages:

1. **Precise discrimination of closely spaced rows.** For complex cases with extremely narrow inter-row spacing, the algorithm utilizes row discrimination strategies to clearly distinguish adjacent rows in most scenarios, overcoming the limitations of traditional approach for close-row discrimination.
2. **Comprehensive handling of complex row morphologies.**
  a. **Partial row precise detection**: The algorithm optimizes recognition logic to extract complete row information from partial features, achieving high-accuracy detection in partial row scenarios and ensuring no missed or false detections.
  b. **Dynamic fitting of curved rows**: For curved rows with varying radii, the algorithm integrates morphological analysis, enabling accurate recognition of diverse field planting configurations.
3. **Noise filtering for high-quality detection.** Equipped with built-in noise elimination mechanisms, the algorithm efficiently filters noise points during detection, preventing invalid interference from affecting row recognition. This enhances result purity and ensures that each recognition step is based on valid data.

### 4.5 Limitations of straight-line fitting approaches to row detection

Besides unicorn, we also evaluated classical line-fitting algorithms, specifically the Hough transform and RANSAC, and found them unsuitable for identifying corn ear rows. Both techniques assume the presence of continuous edges or dense local features, which fundamentally mismatches the sparse, noisy, and discrete nature of kernel coordinates on a corn ear. The Hough transform, which accumulates votes from collinear pixels, fails because the large distances between kernel centroids prevent the formation of distinct peaks in the parameter space. Consequently, true column alignments are obscured by spurious peaks arising from random point combinations. RANSAC repeatedly samples minimal subsets to find the largest inlier set, yet the lateral looseness and occasional missing kernels along the vertical direction make the inlier threshold inapplicable. A small threshold admits noise as inliers, while a large one merges neighboring columns into a single line, leading to unstable detections.

Furthermore, both algorithms are inherently limited to detecting straight lines. This geometric constraint is a notable weakness, because kernel rows often exhibit non-linear shapes, such as micro-curvatures or S-shapes, due to kernel packing, ear morphology, and imaging projection distortions.

### 4.6 CornPheno enables instant in-situ corn ear phenotyping with smartphone

Integrating CornPheno into the WeChat mini-program has significantly enhanced the convenience and practicality of corn ear phenotyping. Leveraging the extensive user base of WeChat and the accessibility of mobile devices, users can now capture images of corn ears and obtain key phenotypic parameters anytime and anywhere using their smartphones. This integration not only lowers the barrier to phenotyping but also dramatically increases data collection efficiency. More importantly, the computational efficiency of the CornPheno enables immediate, on-device processing of phenotypic traits. Breeders can perform phenotyping directly in the field without the need to transport samples back to the laboratory, saving both time and labor cost. By providing quantitative data in real-time, CornPheno supports more timely and data-driven decision-making, promoting the widespread adoption of phenotyping in practical applications.

### 4.7 Measurement biases between single-view and multi-view corn ear phenotyping

CornPheno demonstrates that single-view imaging is effective for measuring kernels per ear, rows per ear, and kernels per row. However, it exhibits limitations for estimating whole-ear parameters. When imaging one side with incomplete rows, the single-view measurement can systematically underestimate the true row count. Similarly, single-view estimation of total kernel count has also inherent bias risks. Irregular kernel morphology or tightly packed arrangements further amplify occlusion errors. Although one could apply an empirical coefficient to extrapolate whole-ear values, the coefficient may be sensitive to per-ear morphology. Achieving high-precision measurements of total kernel and row counts would necessitate multi-view imaging that integrates data from multiple viewpoints.

Despite these limitations, single-view imaging remains valuable in high-throughput phenotyping. The low equipment cost, fast data acquisition, and reduced analytical complexity demonstrates its value for large-scale, preliminary screening of breeding populations. Our smartphone-based single-view framework not only provides an accessible tool for this purpose but also serves as an essential technological foundation upon which more complex, multi-view phenotyping systems can be developed.

### 4.8 Practical use guide

To ensure reliable measurements and high-quality data acquisition in diverse field environments for CornPheno, standardized operational procedures are essential. Prior to image acquisition, a visual inspection should confirm the visibility of dorsal kernel surfaces. For lighting control, uniform diffuse lighting (e.g. overcast conditions or shaded shelters) is prioritized during data collection, which effectively mitigates specular reflections and shadow artifacts from direct sunlight. Backlit shooting and strong contre-jour scenarios should be avoided. Regarding kernel occlusion management, maximizing exposure of geometrically ordered kernel surfaces while minimizing visual obstructions from husk leaves or cob structures is critical.

### 4.9 Efficiency evaluation

To quantify the efficiency of CornPheno, we compare it against the traditional physical counting of kernels on an ear and the manual annotation of corn images. According to breeders, the average time required to count the kernels on a corn cob is approximately three to 4 ​min in practical breeding operations. Based on this, we further carried out an experiment by manually annotating corn images, which revealed that it takes an average of one to 2 ​min to complete the annotation. In contrast, our WeChat-based mini-program requires only four to 5 ​s to analyze a single image, without the need for additional human intervention. Considering that a typical corn cob usually requires two to three images to ensure comprehensive analysis, the total phenotypic analysis time for a single corn cob using our automated system is approximately 15 ​s. Compared with the 180 ∼ 240 ​s required by manual phenotyping, CornPheno offers an approximate 15 times increase in throughput, significantly reducing the time and labor required for corn ear phenotyping.

### 4.10 Limitations and future directions

**Fig. 12:**
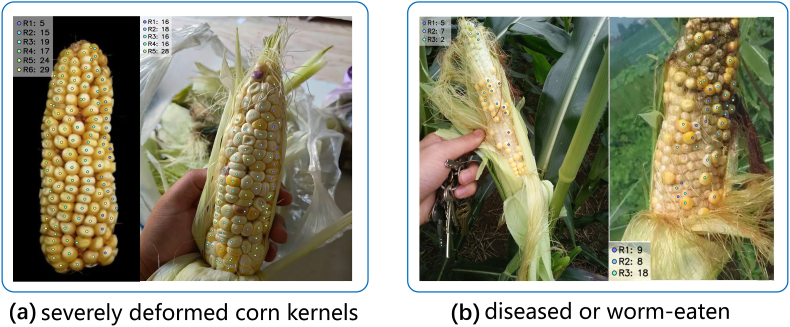
Failure cases of corn ear with diseased, worm-eaten, and deformed kernels.

When dealing with corn ears with few missing kernels, CornPheno maintains good robustness in the detection of kernels per ear, rows per ear, and kernels per row. For corn ears with severe deformation, kernel localization still works well, but the detection of rows per ear and kernels per row shows poor robustness, as shown in Fig. 12a, due to blurred row boundaries. For corn ears with severe missing kernels caused by diseases or insect damage, CornPheno can fail to phenotype the three traits, as shown in Fig. 12b. One possible reason is the dataset bias, as our training data consists mostly of healthy, well-formed ears. Future work will focus on expanding the training dataset with a wider variety of abnormal samples to enhance the generalization of the CornPheno. When tackling corn ears with disordered kernel arrangement, the detection performance of unicorn degrades. This occurs because effort used to maximize point detection can also cause an increase of false positive rates. Consequently, identifying the optimal trade-off between high detection rates and low false positive rates necessitates further studies. This balance is directly tied to the manually set parameter *θ*_*T*_, where distinct datasets inherently require specific *θ*_*T*_ values for optimal adaptation. Future study may focus on developing adaptive or learnable configurations of *θ*_*T*_ capable of accommodating diverse corn cultivars.

## 5 Conclusion

In this work, we propose CornPheno, a user-friendly phenotyping approach that can work in an open environment with a mobile device platform. Users can take photos of corn ears in the field with their smartphones and upload them to the mini-program, then instantly obtaining phenotypic parameters of kernels per ear, rows per ear, and kernels per row. CornPheno features two techniques: CornPET for kernel localization and unicorn for robust row detection. Additionally, we introduce the CKC-Wild dataset, a new benchmark with corn images captured in machine, indoor, and field environments, reflecting real-world challenges.

Extensive experiments on the CKC-Wild dataset show that: i) CornPET achieves the lowest MAE and best *R*^2^; ii) CornPheno performs well under natural lighting and complex backgrounds; iii) unicorn effectively addresses challenges such missing kernels in rows, row distortion, and misalignment, enabling reliable row detection and ear-level kernel counting.

By integrating CornPheno into a WeChat mini-program, breeders can directly capture and upload images of in-situ corn ears without the need for specialized equipment or complex tools, thereby enhancing the convenience and accessibility of phenotypic analysis. This helps agricultural practitioners and researchers to improve their work efficiency and to accelerate decision-making processes.

## CRediT authorship contribution statement

**Xin Li**: Conceptualization, Investigation, Methodology, Validation, Visualization, Writing - original draft, Writing - review & editing.

**Pinzhe Li**: Conceptualization, Investigation, Methodology, Validation, Visualization, Writing - original draft, Writing - review & editing.

**Xinzhe Wang**: Conceptualization, Investigation, Methodology, Validation, Visualization, Writing - original draft, Writing - review & editing.

**Yaning Zhu**: Conceptualization, Investigation, Methodology, Validation, Visualization, Writing - original draft, Writing - review & editing.

**Tianqi Hu**: Conceptualization, Investigation, Methodology, Software, Writing - original draft, Writing - review & editing.

**Yongshuai Zhang**: Software, Writing - review & editing.

**Zhiguo Han**: Supervision, Funding, Writing - review & editing.

**Hao Lu**: Supervision, Conceptualization, Writing - review & editing.

## Declaration of competing interest

The authors declare that they have no known competing financial interests or personal relationships that could have appeared to influence the work reported in this paper.

The author is an Editorial Board Member/Editor-in-Chief/Associate Editor/Guest Editor for this journal and was not involved in the editorial review or the decision to publish this article.

## Data Availability

Data are available upon request.
